# Wettability and confinement size effects on stability of water conveying nanotubes

**DOI:** 10.1038/s41598-020-74398-x

**Published:** 2020-10-13

**Authors:** M. Shaat, U. Javed, S. Faroughi

**Affiliations:** 1 Mechanical Engineering Department, Abu Dhabi University, P.O.BOX 1790, Al Ain, United Arab Emirates; 2 Department of Engineering, American University of Iraq Sulaimani (AUIS), Sulaimania, 46001 Iraq; 3 Faculty of Mechanical Engineering, Urmia University of Technology, Urmia, Iran

**Keywords:** Carbon nanotubes and fullerenes, Phase transitions and critical phenomena, Fluid dynamics

## Abstract

This study investigates the wettability and confinement size effects on vibration and stability of water conveying nanotubes. We present an accurate assessment of nanotube stability by considering the exact mechanics of the fluid that is confined in the nanotube. Information on the stability of nanotubes in relation to the fluid viscosity, the driving force of the fluid flow, the surface wettability of the nanotube, and the nanotube size is missing in the literature. For the first time, we explore the surface wettability dependence of the nanotube natural frequencies and stability. By means of hybrid continuum-molecular mechanics (HCMM), we determined water viscosity variations inside the nanotube. Nanotubes with different surface wettability varying from super-hydrophobic to super-hydrophilic nanotubes were studied. We demonstrated a multiphase structure of nanoconfined water in nanotubes. Water was seen as vapor at the interface with the nanotube, ice shell in the middle, and liquid water in the nanotube core. The average velocity of water flow in the nanotube was obtained strongly depend on the surface wettability and the confinement size. In addition, we report the natural frequencies of the nanotube as functions of the applied pressure and the nanotube size. Mode divergence and flutter instabilities were observed, and the activation of these instabilities strongly depended on the nanotube surface wettability and size. This work gives important insights into understanding the stability of nanotubes conveying fluids depending on the operating pressures and the wettability and size of confinement. We revealed that hydrophilic nanotubes are generally more stable than hydrophobic nanotubes when conveying fluids.

## Introduction

With the advancement of nanotechnology, nanotube making has been considerably flourished in the past few years. Nanotubes can be made with perfect hollow cylindrical geometries and superior mechanical, chemical and thermal stabilities. The currently developed nano-biological and nano-mechanical systems mainly depend on nanotubes for fluids’ storage and fluids’ transport^1–5^. The design of these nano-scale systems requires investigations on the mechanics of the fluid in the nanotube and predictions of the nanotube stability under different fluid flow rates.

Studies were carried out on the stability of nanotubes conveying fluids^4,6–17^. The influence of the fluid flow on the free vibration of nanotubes has been investigated using linear models of the nanotube dynamics^11–14,18–20^. These linear models were used to determine the onsets of the fluid flow-induced instability of nanotubes under different conditions^6,10,15,21–24^. For example, the free vibration and instability onset of water conveying single-walled carbon nanotubes (CNTs) with cantilever, simply supported, and clamped–clamped boundary conditions have been studied^11,13,15^. Dong et al.^10^ investigated the wave propagation in multi-walled CNTs embedded in an elastic medium. Tang et al.^7,16,17^ studied the divergence instability of nonhomogeneous nanotubes conveying fluids. These studies revealed that the frequencies of the free vibration of nanotubes conveying fluids decrease as the fluid flow velocity increases^7,11–13,16,17^. It was also demonstrated that the divergence instability of a particular mode would be triggered if the fluid flow exceeds a critical velocity value^6,13,14,25–27^. Critical velocity values were also determined at which a particular mode would be coupled with a successive mode, and a coupled-mode flutter would occur^13,14,27^. Other studies have implemented nonlinear models to reveal the behavior of the nanotube under the instabilities due to the fluid flow^28,29^.

The aforementioned studies have explored the influence of the fluid flow on the dynamics of nanotubes. Nonetheless, these studies were carried out with no consideration for the exact mechanics of the fluid that is severely confined in the nanotube. Recent investigations on the mechanics of fluids confined in nanostructures revealed unusual fluid characteristics and behaviors^30–41^. For example, flow rates of nanoconfined water in CNTs were measured to be several orders-of-magnitude higher than the predicted flow rates by the classical Hagen-Poiseuille equation for bulk water in macroscopic tubes^36,37,39,42,43^. This has been attributed to the fluid slip over the surface of the confining tube^34,44,45^. Therefore, the dynamics of nanotubes conveying fluids has been studied based on measures of the fluid slip boundary conditions^27,46–50^. However, these studies still hide many other important measures of the nanoconfined fluid mechanics. For example, nanoconfined water was observed sticking to or, even, seeping through a super-hydrophilic surface^32^. Moreover, multiphase structures were revealed for some fluids under severe nanoconfinement conditions at hydrophobic and hydrophilic interfaces^51–55^. Thus, a liquid can easily morph into solid and/or gas when it is nanoconfined^30,31,34,53,55–61^. The aforementioned nontraditional phenomena of nanoconfined fluids would robustly influence the dynamics and stability of the nanotube. Therefore, accurate predictions of the dynamics and stability of nanotubes conveying fluids should be carried out accounting for all these nontraditional phenomena.

In this study, we put the stability of water conveying nanotubes under scrutiny accounting for the effects of the nanotube size and the wettability of its surface. When water is inside nanoscale conveyers, many important parameters come into play, namely wettability of the confining surface, water–surface interactions, and confinement size^34,62^. Previous studies gave no consideration to these parameters and their effects on the frequencies and stability of fluid conveying nanotubes. This study fills into this gap, and the frequency variations as functions of the nanotube surface wettability and the nanotube diameter are determined. First, the exact mechanics of nanoconfined water in comparison to the mechanics of bulk water are investigated. Then, the free vibration of the nanotube is analytically solved. Numerical results are also represented to explore the wettability and the confinement size effects on the frequencies and stability of water conveying nanotubes.

It should be mentioned that the present study captures the dynamics and the stability of water conveying nanotubes similar to the studies mentioned above. The difference, though, resides in the fact that an accurate and appropriate model of the exact mechanics of the nanoconfined fluid is used in conjunction with the equation of solid mechanics to investigate the dynamics of water conveying nanotubes more accurately than before. This enables us to investigate the wettability and the confinement size effects on the dynamics and stability of water conveying nanotubes.

## Mechanics of nanoconfined water

Water exhibits many nontraditional phenomena when it is nanoconfined. Water flow would be enhanced or inhibited when it is driven to flow in nanoslits or nanotubes^36,37,39,42,43,45,53^. Enhanced flow rates were measured when nanoconfined water flow adjacent to hydrophobic surfaces^34,44,45,62,63^ and hydrophilic surfaces^45,60,64,65^. Adjacent to a superhydrophilic surface, water particles were observed sticking to the surface, and a significant flow inhibition was detected^32^. These observations indicated that the characteristics of nanoconfined water are drastically different from those of bulk water^30,32,37,38,66^. For instance, the equivalent viscosity of nanoconfined water was determined lower than the one of bulk water by $\sim 56.5\%$ when flowed in a CNT with $\sim 1.4$ nm diameter^34^.

**Figure 1:**
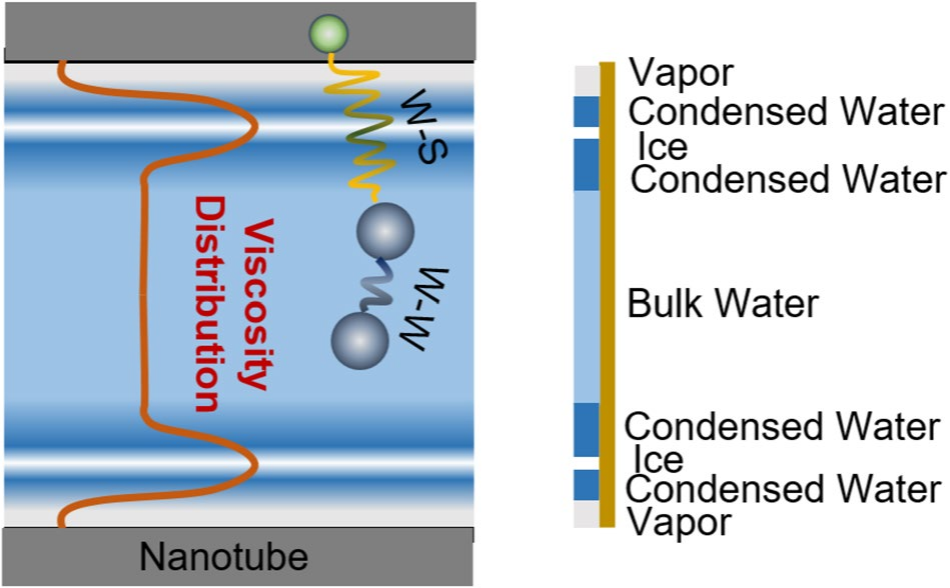
Multiphase structure of water nanoconfined in a nanotube. Water phase changes from vapor at the interface to condensed water and ice at the first water layer. Then, it changes to bulk water at the nanotube center. A schematic of the distribution of water viscosity through the nanotube radial direction is shown. W–W refers to water–water interactions while W–S refers to water–solid interactions.

Nanoconfined water exhibits a multiphase structure under ambient conditions^51–55^. A typical schematic of a multiphase structure of water in a nanotube is shown in Fig. 1. In hydrophobic (and some hydrophilic) nanotubes, water particles would be depleted from a thin layer adjacent to the tube surface. The depletion layer is characterized by an intensive decrease in the water density^34,37,45,55^. Surpassing the depletion layer and at the first water layer, the density sharply increases, and this behavior follows a radial distribution towards the nanotube center^32,34,39,45^. The radial variations of the water density were attributed to water–surface interactions, which are promoted by the nanoconfinement of water^34,55^. Based on water density variations, multiphase structures of water in nanotubes were observed^51–54^. Early studies demonstrated and shed light on the phase transitions of nanoconfined water into vapor and/or ice^30,31,53,56,57,60,61,65^. In these studies, phase transitions were demonstrated based on the density variations upon the severe confinement of water.

When it comes to investigations of multiphase structures and phase transitions of water based on the viscosity variations, there has been a gap for quite a while, and therefore studies on variations of water viscosity between the confining surfaces were much needed. It is well known that viscosity quantifies the fluidity and describes the continuity of molecular interactions of the continuum. For design purposes of nanofluidics, information on the viscosity and how it would be affected by the confining conditions are needed.

**Figure 2:**
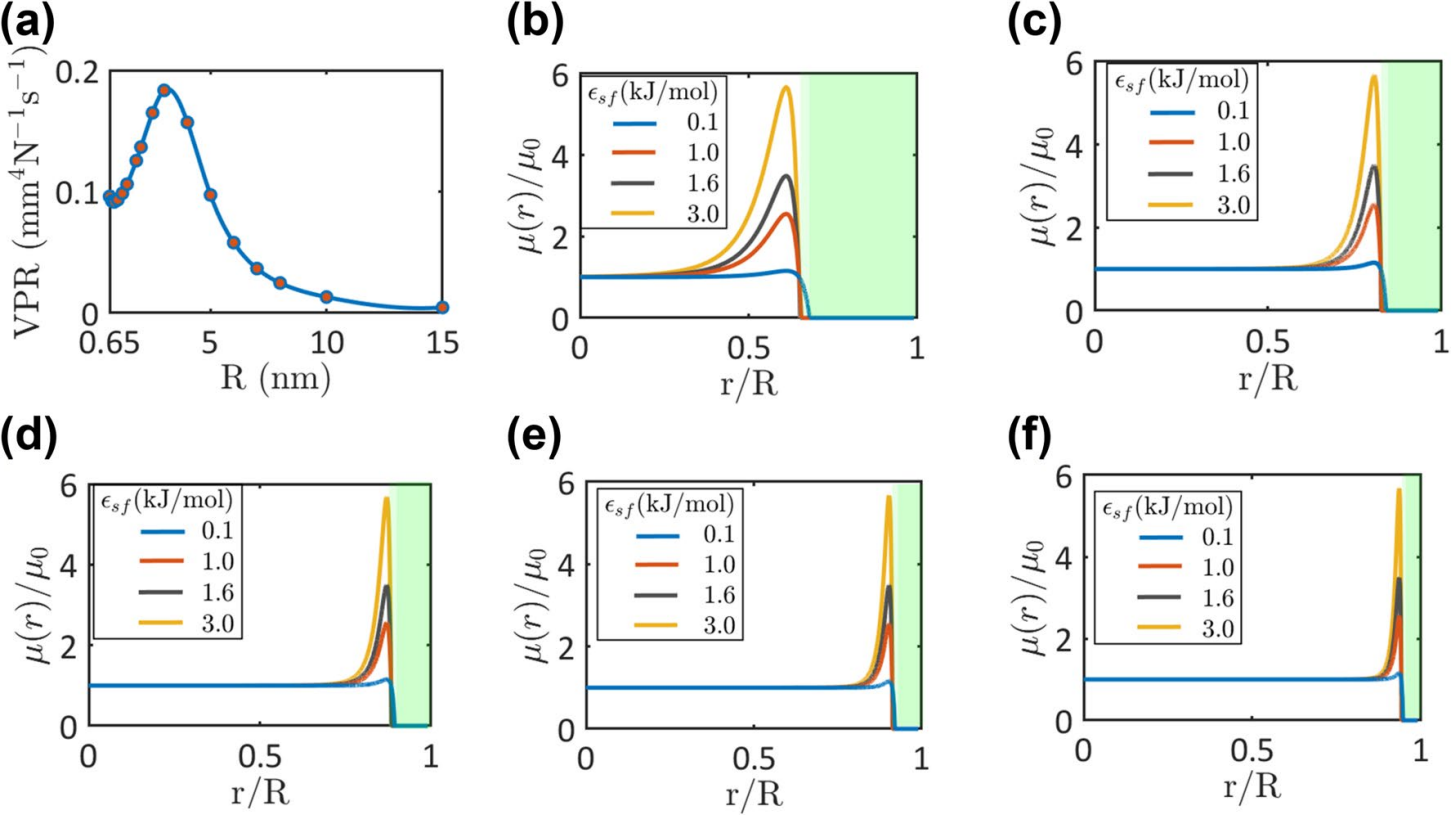
(**a**) The slip velocity to pressure gradient ratio as a function of the nanotube radius (VPR function)^34,55^. (**b**–**f**) Radial distributions of the viscosity of nanoconfined water in nanotubes with (**b**) *R* = 1 nm, (**c**) *R* = 2 nm, (**d**) *R* = 3 nm, (**e**) *R* = 4 nm, and (**f**) *R* = 6 nm. The viscosity distributions are represented for different values of the water–surface interaction energy ($\epsilon_{sf} = 0.1,\;1,\;1.6, \;{\text{and}}\; 3$ kJ/mol). The green highlights indicate water–nanotube interface.

Shaat and Zheng^55^ developed a hybrid continuum-molecular mechanics (HCMM) approach to report the distribution of water viscosity between two confining surfaces. They revealed that the interfacial viscosity of water in a nanotube ($\mu_{I}$) (the viscosity of water at the interface with the nanotube surface) strongly depends on the wettability of the nanotube surface and the nanotube diameter, and it is totally different than the viscosity elsewhere. The interfacial viscosity was expressed in terms of a *slip velocity-to-pressure gradient ratio* (VPR), which was determined based on molecular dynamics simulations and experimental measurements of water flow in different nanotubes^55^. In addition, it was demonstrated that water viscosity sharply increases at the first water layer due to water–surface interactions. The rise of the viscosity at this layer depended on the relative hydrodynamics of the nanoconfined water and its bulk counterpart. Because the viscosity and the hydrodynamics of water depend on the interatomic potential, water’s core viscosity was defined using the ratio of the fluid–fluid interatomic force to the fluid–surface interatomic force^55^. Given these observations, the radial variation of the water viscosity was defined to represent the multiphase structure of the nanoconfined water in a nanotube, as follows^55^:

1 $$\mu \left( r \right) = \left\{ {\begin{array}{*{20}l} {\frac{{2.355\left( {R^{2} - \left( {R - \delta } \right)^{2} } \right)}}{{{\text{VPR}}\left( R \right)}}} \hfill & { \quad R - \delta \le r \le R} \hfill \\ {\mu_{0} \left( {1 - \frac{{5\epsilon_{sf} \sigma_{ff} }}{{3\epsilon_{ff} \sigma_{sf} }}\left[ {12\left( {\frac{{\sigma_{sf} }}{R - r}} \right)^{13} - 6\left( {\frac{{\sigma_{sf} }}{R - r}} \right)^{7} } \right]} \right)} \hfill & {\quad 0 < r < R - \delta } \hfill \\ \end{array} } \right.$$

where $R$ is the nanotube’s inner radius, and $\delta$ is the interface thickness (for fluids in nanotubes $\delta = 1.1224 \sigma_{sf}$^34^). $\sigma_{sf}$ and $\epsilon_{sf}$ are constants of Lennard–Jones (*LJ*) potential of water–surface interactions, and $\epsilon_{ff}$ and $\sigma_{ff}$ are constants of LJ potential of water–water interactions (see Fig. 1). $\mu_{0}$ is the viscosity of bulk water. $r$ is the radial-coordinate of the nanotube. Figure 2a shows the VPR function as determined by Shaat^34,55^.

Figure 2b–f show the radial distribution of water viscosity in different nanotubes with different surface wettability and diameters. The wettability of the nanotube changes depending on water–surface interaction energy $\epsilon_{sf}$, as follows:

2 $$\theta \left( {{\text{rad}}} \right) = - 0.97\epsilon_{sf} \left( {{\text{kJ}}/{\text{mol}}} \right) + \pi$$

where $\theta$ is the contact angle, which is a measure of the surface wettability. Thus, the nanotube is hydrophobic (i.e., $\theta > \pi /2$) when $\epsilon_{sf} < \sim 1.6$ kJ/mol and hydrophilic (i.e., $\theta < \pi /2$) when $\epsilon_{sf} > \sim 1.6$ kJ/mol. To investigate the surface wettability effects on water viscosity, results were depicted when changing the wettability between super-hydrophobic nanotubes ($\epsilon_{sf} = 0.1$ kJ/mol) and super-hydrophilic nanotubes ($\epsilon_{sf} = 3$ kJ/mol). In addition, various nanotubes of different diameters were simulated to reveal the confinement size effects on water viscosity.

At the interface with the nanotube (green highlighted regions in Fig. 2), water is depleted and its viscosity is significantly decreased. On the other hand, water accumulate the region just beyond the interface (commonly known as the first water layer) and the viscosity is sharply increased. This can be attributed to the interaction energy between water particles and particles of the nanotube surface, which radially varies inside the nanotube. Water–surface interaction is repulsion very close to the nanotube surface (at the interface) and attraction elsewhere. Therefore, water is depleted at the interface, but it accumulates layers after the depletion region. The intensity of the attraction energy is maximum at the first water layer, and it decreases towards the nanotube center.

It follows from Fig. 2b–f that an increase in the water–surface interaction energy ($\epsilon_{sf}$) is accompanied with an increase in the viscosity at the first water layer. Water viscosity is $\sim 6$ times the viscosity of bulk water when water is confined in a super-hydrophilic surface ($\epsilon_{sf} = 3$ kJ/mol). Whereas the viscosity intensively decreases at the interface, the increase in the viscosity at the first water layer would lead to an equivalent water viscosity that is generally higher than the bulk water viscosity. This indicates that water would be more viscous when it is confined in hydrophilic nanotubes. On the other hand, the viscosity at the first water layer is almost the same as the bulk water when it is nanoconfined in a super-hydrophobic surface ($\epsilon_{sf} = 0.1$ kJ/mol). In contrast to hydrophilic nanotubes, water would be inviscid when it is confined in hydrophobic nanotubes.

Figure 2b–f reveal the confinement size effects on water viscosity. As the nanotube size decreases, the interfacial region increases, which is featured with a reduced viscosity. This indicates that the equivalent water viscosity decreases as the nanotube size decreases. This can be mainly attributed to the increase in the contribution of the water–surface interaction energy to water’s mechanics due to a decrease in the nanotube size.

## Mechanics of fluid conveying nanotubes

The equation that governs the linear dynamics of a nanotube conveying a fluid that flows at an average flow velocity $V$ can be written, as follows^67^:

3 $$EI\frac{{\partial^{4} w\left( {x,t} \right)}}{{\partial x^{4} }} + m_{f} V^{2} \frac{{\partial^{2} w\left( {x,t} \right)}}{{\partial x^{2} }} + 2m_{f} V\frac{{\partial^{2} w\left( {x,t} \right)}}{\partial x\partial t} + \left( {m_{s} + m_{f} } \right)\frac{{\partial^{2} w\left( {x,t} \right)}}{{\partial t^{2} }} = 0$$

where $EI$ is the flexural stiffness of the nanotube, and $w\left( {x,t} \right)$ is the nanotube fluid flow-induced deflection. $m_{f}$ is the mass per unit length of the fluid, and $m_{s}$ is the mass per unit length of the nanotube. $V$ is the average velocity of the fluid’s steady state flow. $x$ and $t$ are the axial coordinate and time, respectively.

The equation of motion (3) was derived assuming a steady state laminar flow of a Newtonian fluid, and the nanotube is Euler–Bernoulli beam. This linear equation of motion can be used to determine the *onsets* of the various instabilities of the nanotube due to the fluid flow. The onsets of these instabilities can be defined by bifurcation points of the eigenvalues-fluid velocity curves in the complex Argand plane^67^. However, the mode-shapes or the nanotube’s behavior under the various instability conditions would require a nonlinear model that accounts for the possible axial stretching of the nanotube.

The dynamic damping of the nanotube is neglected in Eq. (3). Nanotubes-mechanical resonators have revealed exceptional quality factors exceeding five million^68^. In addition, the fluid flows inside the nanotube with a nearly-zero friction due to the severe confinement and its phase transition to gas at the fluid–nanotube interface^69,70^. Therefore, the dynamic damping effect is omitted from Eq. (3) resulting in a conservative system of a slender nanotube conveying fluid. Such a system is expected to exhibit static divergence instability and Hamiltonian flutter instability as the fluid flow velocity increases^67^.

The left side of the equation constitutes four terms that are the stiffness, centrifugal, Coriolis or gyroscopic, and inertial forces, respectively. The centrifugal and Coriolis terms (2nd and 3rd terms in Eq. (3)) depend on the momentum of the fluid flow in the nanotube, which mainly depends on the fluid viscosity. We previously demonstrated that the fluid viscosity would significantly change when it is used under severe confinement conditions. Therefore, investigations on the stability of nanotubes conveying fluids should be carried out with a careful consideration of the effects of the confinement conditions on the fluid mechanics. Whereas the stability of nanotubes conveying fluids has been investigated in previous studies^4,6,10–15^, it was challenging to relate the nanotube dynamics to the fluid viscosity. The dynamics and stability of nanotubes conveying fluids were investigated based on the fluid’s dynamics. However, the practical use of these nanosystems requires reports on the nanotube dynamics and stability in relation to the applied pressure, fluid viscosity, nanotube size, and nanotube-surface wettability. Here, we provide these relations and report the stability of nanotubes depending on these factors, which are missing in previous studies.

To reveal the stability of nanotubes conveying water in relation to the applied pressure, fluid viscosity, nanotube size and nanotube-surface wettability, the traditional Hagen–Poiseuille model of pressure-driven water flow in circular tubes is modified based on the new mechanics of nanoconfined water that is explained in “Mechanics of nanoconfined water” section. Utilizing the viscosity function $\mu \left( r \right)$ [Eq. (1)], Hagen–Poiseuille model was modified and the velocity profile of water flow in nanotubes was obtained in the form^55^:

4 $$v\left( r \right) = \frac{P}{2L}\left( {\left( {\smallint \frac{r}{\mu \left( r \right)}dr} \right)_{r = R} - \smallint \frac{r}{\mu \left( r \right)}dr} \right)$$

where $v\left( r \right)$ is the fluid’s velocity function, $P$ is the applied pressure (pressure drop at the nanotube ends), and $L$ is the nanotube length. The average velocity, $V$, can be then determined as follows:

5 $$V= \frac{1}{{\pi R^{2} }}\mathop \smallint \limits_{0}^{R} 2\pi rv\left( r \right)dr = \frac{P}{2L}\left( {\smallint \frac{r}{\mu \left( r \right)}dr} \right)_{r = R} - \frac{P}{{R^{2} L}}\mathop \smallint \limits_{0}^{R} \left( {\smallint \frac{r}{\mu \left( r \right)}dr} \right)rdr$$

The substitution of Eq. (5) into Eq. (3) gives the equation of motion of the nanotube conveying fluid depends on the applied pressure, fluid viscosity, nanotube size, and nanotube-surface wettability.

## Analytical solution

Here, the eigenvalue problem of the free vibration of nanotubes conveying water is analytically solved. First, the equation of motion [Eq. (3)] is rewritten employing the following nondimensional parameters:

6 $$X = \frac{x}{L};\;W\left( X \right) = w\left( x \right)\sqrt {\frac{A}{I}} ;\;T = t\sqrt {\frac{{EI}}{{\left( {m_{f} + m_{s} } \right)L^{4} }}}$$

The substitution of Eq. (6) into Eq. (3) gives:

7 $$\frac{{\partial^{4} W\left( {X,T} \right)}}{{\partial X^{4} }} + \beta_{1}^{2} \frac{{\partial^{2} W\left( {X,T} \right)}}{{\partial X^{2} }} + \beta_{2}^{2} \frac{{\partial^{2} W\left( {X,T} \right)}}{\partial X\partial T} + \frac{{\partial^{2} W\left( {X,T} \right)}}{{\partial T^{2} }} = 0$$

where

8 $$\beta_{1} = \sqrt {\frac{{m_{f} V^{2} L^{2} }}{EI}} ;\;\beta_{2} = \sqrt {\frac{{2m_{f} VL}}{{\left( {m_{s} + m_{f} } \right)EI}}}$$

The deflection can be decomposed as follows:

9 $$W\left( {X,T} \right) = \varphi \left( X \right)\exp \left( {i\omega T} \right)$$

where $\varphi \left( X \right)$ is the mode shape function, and $\lambda$ is the eigenvalue. $\omega$ is the nondimensional natural frequency. By substituting $\varphi \left( X \right) = \exp \left( {i\lambda X} \right)$ into Eq. (9) and substituting the result into Eq. (7), the following characteristics equation is obtained:

10 $$\lambda^{4} - \beta_{1}^{2} \lambda^{2} - \omega \beta_{2}^{2} \lambda - \omega^{2} = 0$$

For simply supported nanotubes, the following boundary conditions are applied:

11 $$\begin{aligned} & \varphi \left( 0 \right) = \varphi \left( 1 \right) = 0 \\ & \frac{{d^{2} \varphi \left( 0 \right)}}{{dX^{2} }} = \frac{{d^{2} \varphi \left( 1 \right)}}{{dX^{2} }} = 0 \\ \end{aligned}$$

Accordingly, the nondimensional natural frequencies can be determined by solving the following equation:

12 $$\det \left[ {\begin{array}{*{20}c} 1 & 1 & 1 & 1 \\ {\lambda_{1}^{2} } & {\lambda_{2}^{2} } & {\lambda_{3}^{2} } & {\lambda_{4}^{2} } \\ {\exp \left( {i\lambda_{1} } \right)} & {\exp \left( {i\lambda_{2} } \right)} & {\exp \left( {i\lambda_{3} } \right)} & {\exp \left( {i\lambda_{4} } \right)} \\ {\lambda_{1}^{2} \exp \left( {i\lambda_{1} } \right)} & {\lambda_{2}^{2} \exp \left( {i\lambda_{2} } \right)} & {\lambda_{3}^{2} \exp \left( {i\lambda_{3} } \right)} & {\lambda_{4}^{2} \exp \left( {i\lambda_{4} } \right)} \\ \end{array} } \right] = 0$$

where $\lambda_{r}$ (i.e., $r = 1 \to 4$) are the roots of the polynomial in Eq. (10).

It should be mentioned that the determinant relation in Eq. (12) is complex, and the natural frequencies are determined based on its real part. In the present study, a procedure is employed to determine the natural frequencies of the nanotube. In this procedure, the real part of the determinant relation is plotted as a function of the frequency, $\omega$. Thus, a wide range of frequencies is initially assumed. Then, a value of the frequency is substituted into Eq. (10) and the roots, $\lambda_{r}$, are obtained. The roots $\lambda_{r}$ are then substituted into Eq. (12), and the determinant is calculated. The process is repeated over the assumed range of the frequencies, and the obtained determinant values are plotted against the frequency, $\omega$. The natural frequencies of the nanotube are determined by intersecting the plotted curve with the zero determinant.

## Results and discussions

Here, effects of the applied pressure, fluid viscosity, nanotube size, and nanotube surface wettability on the dynamics and stability of nanotubes conveying fluids are investigated. Results were extracted to depict the evolution of the nondimensional natural frequencies of the nanotube with the increase in the applied pressure ($P = 0 \to 30$ GPa), water–surface interaction energy ($\epsilon_{sf} = 0.1 \to 3$ kJ/mol), and nanotube size ($R = 0.7 \to 15$ nm). The material and geometrical parameters as considered in the presented results are given in Table 1. A CNT with an elastic modulus of 358.1 GPa was considered. Despite the elastic properties of nanomaterials are generally size-dependents, the size-dependence of the elastic modulus of CNT is negligible^71–73^. For example, the molecular dynamics simulations of the evolution of the elastic modulus of CNTs with the size decrease indicated a decrease in the elastic modulus from 360 to 320 GPa when the nanotube radius was decreased from 1 nm to 0.2 nm^73^. CNTs with radii bigger than 1 nm gave no size dependence of the elastic modulus. Therefore, here, the same material properties of the CNT are considered for the entire nanotube radius range.

**Table 1:** Material and geometrical parameters of the considered water conveying nanotube system.

Parameter	Value
Nanotube*	
Inner radius, R (nm)	$0.7 \to 15$
Wall-thickness, $h$ (nm)	0.34
Length, $L$ (nm)	100
Young’s modulus, $E$ (GPa)^71^	358.1
Mass density, $\rho_{s}$ (kg/m^3^)^71^	2266
**Water**	
Lennard–Jones parameters^55^	
$\sigma_{ff}$(nm)	0.3169
$\epsilon_{ff}$(kJ/mol)	0.651
Bulk water viscosity, $\mu_{0}$ (Pa.s)	0.001
Water density, $\rho_{f}$ (kg/m^3^)	1000
**Water–nanotube interactions**	
$\sigma_{sf}$(nm)	0.3122
$\epsilon_{sf}$(kJ/mol)	$\epsilon_{sf} = 0.1 \to 3$ kJ/mol

*The mass and inertia parameters, $m_{f}$, $m_{s}$, and $I$ introduced in Eq. (3) are calculated for the considered water conveying nanotube system, as follows: $m_{f} = \pi R^{2} \rho_{f}$; $m_{s} = \pi \left( {\left( {R + h} \right)^{2} - R^{2} } \right)\rho_{s}$; $I = \frac{\pi }{4}\left( {\left( {R + h} \right)^{4} - R^{4} } \right)$.

A verification of the proposed model was carried out. First, the HCMM, which is used to determine water viscosity [Eq. (1)], was verified by the comparison with over 90 cases of experiments and molecular dynamics simulations of water flow in nanotubes^55^. In addition, the proposed model of the free vibration of the nanotube conveying fluid was verified by the comparison with the results of fluid flow in CNTs in the literature^74^ (see Supplementary Information).

**Figure 3:**
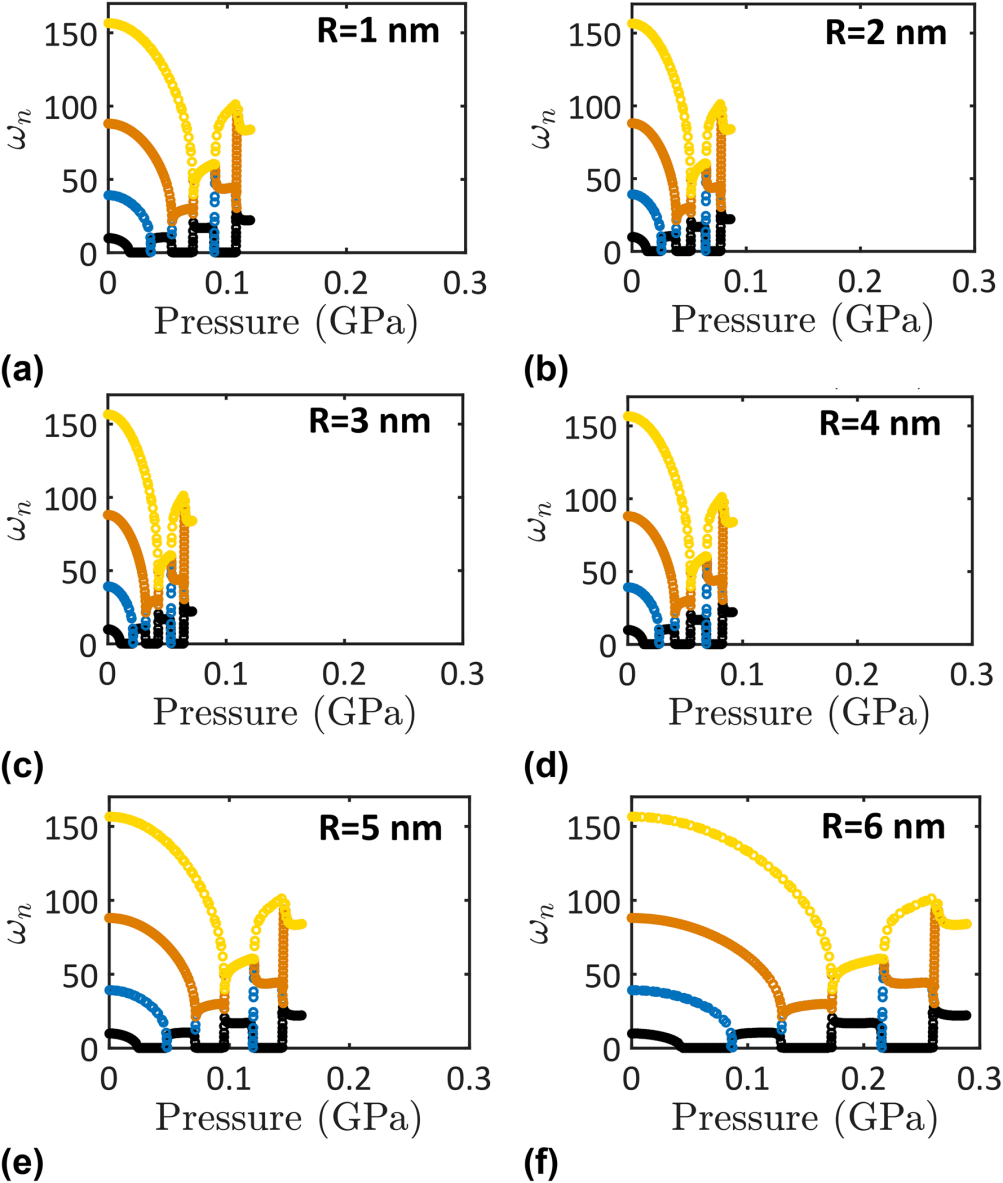
The first four nondimensional natural frequencies as functions of the applied pressure of hydrophobic nanotubes ($\epsilon_{sf} = 0.1$ kJ/mol) with different radii; (**a**) R = 1 nm, (**b**) R = 2 nm, (**c**) R = 3 nm, (**d**) R = 4 nm, (**e**) R = 5 nm, and (**f**) R = 6 nm.

**Figure 4:**
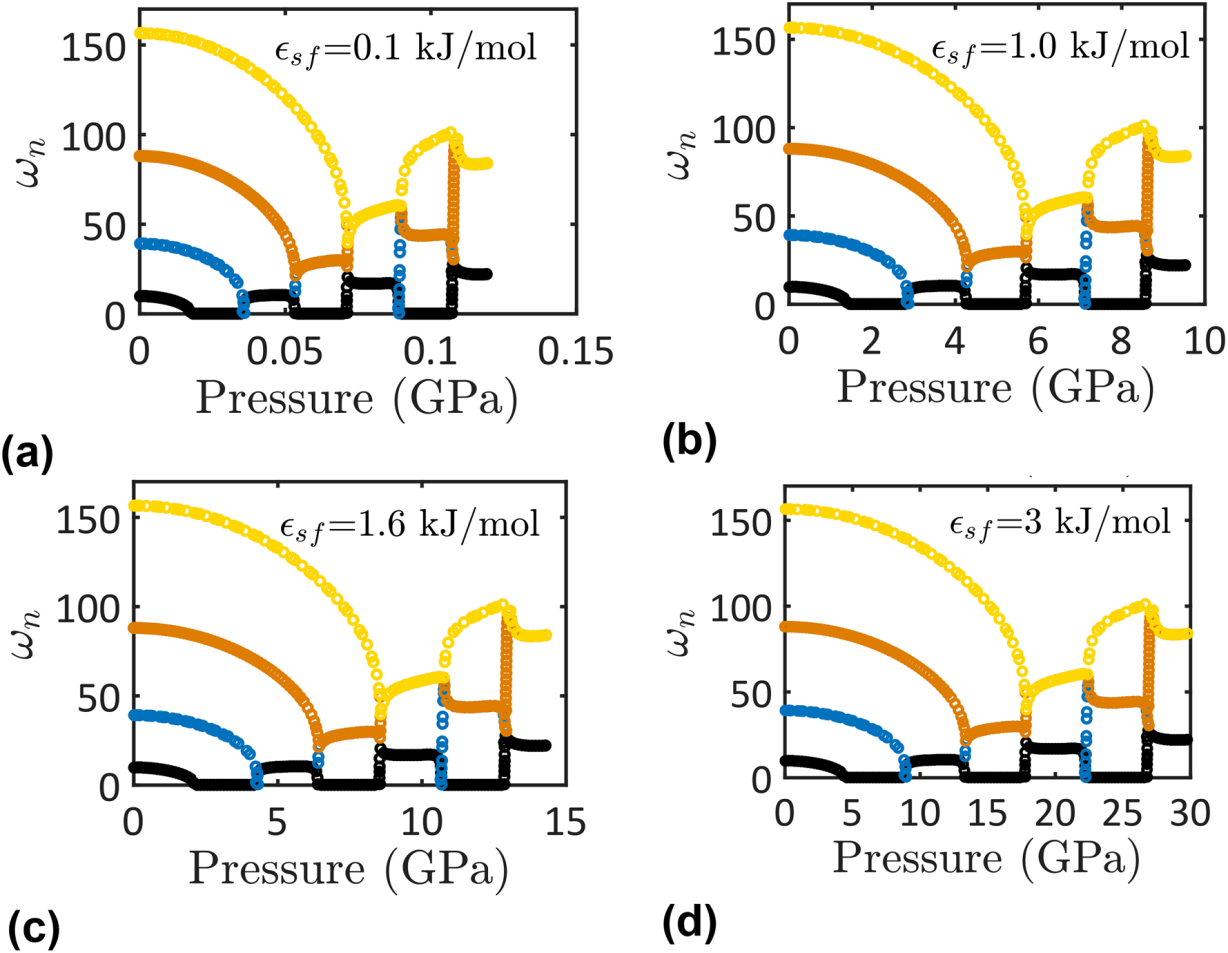
The first four nondimensional natural frequencies as functions of the applied pressure of nanotubes (*R* = 1 nm) with different surface wettability; (**a**) $\epsilon_{sf} = 0.1$ kJ/mol, (**b**) $\epsilon_{sf} = 1.0$ kJ/mol, (**c**) $\epsilon_{sf} = 1.6$ kJ/mol, and (**d**) $\epsilon_{sf} = 3.0$ kJ/mol.

**Figure 5:**
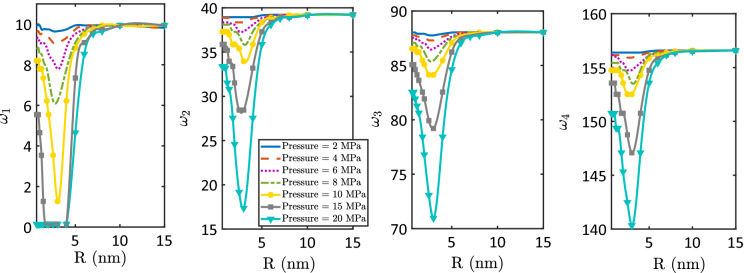
The effect of the confinement size on the first four nondimensional natural frequencies of hydrophobic nanotubes ($\epsilon_{sf} = 0.1$ kJ/mol).

The evolutions of the nondimensional natural frequencies of the first four modes of vibration of nanotubes conveying water with the increase in the applied pressure are depicted in Figs. 3 and 4. In Fig. 3, the natural frequencies of strongly hydrophobic nanotubes ($\epsilon_{sf} = 0.1$ kJ/mol) of different sizes are investigated. The effects of water–surface interactions (or nanotube surface wettability) ($\epsilon_{sf}$) on the natural frequencies of a nanotube of 1 nm radius are depicted in Fig. 4. In addition, the variations of the first four nondimensional natural frequencies of hydrophobic nanotubes ($\epsilon_{sf} = 0.1$ kJ/mol) as functions of the nanotube radius are depicted for different applied pressures in Fig. 5.

According to Fig. 3, the nanotube vibration is classical and independent of its size and surface wettability when water is not pressured to flow (i.e., $P = 0$). Thus, the nondimensional natural frequencies of the first four modes of vibration are the ones of classical simply supported beams (i.e., $\omega_{1} = 9.823$, $\omega_{2} = 39.19$, $\omega_{3} = 88.05$, $\omega_{4} = 156.6$) when $P = 0$. However, upon applying a pressure, water starts to flow at an average velocity that depends on the applied pressure, the nanotube size, and the nanotube surface wettability. Because of the momentum of the flowing water, the natural frequencies of the nanotube are affected and generally decrease lower than the ones of a nanotube with no flowing water. An increase in the applied pressure of fluid is accompanied by a parabolic decrease in the natural frequencies.

Bifurcation points can be defined in Figs. 3 and 4 that indicates the onsets of the various instabilities of the nanotube due to the fluid flow. These bifurcation points were defined by critical velocity values in the previous studies. Here, for the first time, the critical values of the applied pressure at which the various bifurcations of the nanotube conveying fluid occur are defined. Two bifurcations are revealed in Figs. 3 and 4; *pitchfork bifurcation*, where a zero eigenvalue occurs, and *Hamiltonian hopf bifurcation*, where two modes are coupled. The pitchfork bifurcation indicates the onset of a divergence instability while the Hamiltonian hopf bifurcation indicates the onset of a coupled-mode flutter instability.

Beyond a critical value of the applied pressure, the nanotube exhibits different instabilities including mode divergence instability and flutter instability. For instance, the critical pressure for a super-hydrophobic nanotube with R = 6 nm and $\epsilon_{sf} = 0.1$ kJ/mol is 44.4 MPa (Fig. 3). Beyond this value, the nanotube exhibits a first-mode divergence where $\omega_{1} = 0$. As the pressure is further increased, the natural frequency of the second mode decreases down to zero at $P = 129$ MPa indicating a second-mode divergence (Fig. 3). Just beyond the latter pressure value, the nanotube exhibits a coupled-mode flutter where the first and the second modes of vibration are merged. In continue, the divergence and coupling of the higher modes occur as the applied pressure increases. The detection of the critical pressure values at which the different modes would diverge or couple together are important for the practical application of nanotubes conveying water.

Figure 3 depicts the confinement size dependence of the nanotube’s frequencies. It follows from Fig. 3 that the critical pressure value would increase/decrease as the nanotube size decreases. The lowest critical pressure value (this is the pressure value at which the first-mode divergence takes place) decreases from 44.4 to 11 MPa due to a decrease in the nanotube radius from 6 to 3 nm. This trend is then switched when the nanotube radius is further decreased from 3 to 1 nm where the lowest critical pressure slightly increased from 11 to 18.4 MPa.

The surface wettability dependence of the nanotube stability is depicted in Fig. 4. Results presented in Fig. 4 indicate that the critical pressure values would vary depending on the nanotube surface. For instance, the critical pressure values at which the vibrational modes would diverge and/or merge increase as the nanotube hydrophilicity increases. The pressure value at which the first-mode divergence takes place increases from 1.84 to 458.6 MPa due to an increase in the hydrophilicity from a super-hydrophobic nanotube, $\epsilon_{sf} = 0.1$ kJ/mol, to a super-hydrophilic nanotube, $\epsilon_{sf} = 3$ kJ/mol. This means that, under the same pressure levels, hydrophilic nanotubes are more stable than hydrophobic nanotubes. In addition, for applications where operational pressures can change from time to time temporarily or change permanently, hydrophilic nanotubes are best for stable transport of water.

The above qualitative, and to a certain extent quantitative, behavior holds true for all nanotube sizes. Nevertheless, with decrease in the nanotube size, the critical pressure values at which a mode divergence and coupling take place change depending on the nanotube radius (Fig. 3) and the nanotube surface wettability (Fig. 4). This behavior indicates that the pressure dependence of the nanotube stability is unpredictable unless careful calculations are made based on the model developed here.

Figure 5 demonstrates effects of the confinement size on the natural frequencies of nanotubes for different operating pressures from 2 to 20 MPa. It is clear that the nondimensional natural frequencies are as of the ones of a nanotube with no fluid flow when $R \ge 10$ nm. For nanotubes with $R < 10$ nm, the nondimensional natural frequencies vary with a decrease followed by an increase as the nanotube radius decreases. This behavior can be attributed to two mechanisms that differently influence the velocity of water flow in the nanotube. The first mechanism is water flow enhancement due to nanotube size reduction. The decrease in the nanotube size promotes water–surface interactions and the role of the surface wettability. As a result, water is depleted at the interface, and the size of the depletion layer increases as the nanotube size decreases (as previously demonstrated in Fig. 2). Because of the depletion layer, water’s average flow velocity is increased, and the nondimensional frequency is decreased. The other mechanism is the decrease in the average flow velocity due to a decrease in the nanotube size. It is commonly known that, at the same applied pressure, the average flow velocity decreases as the tube size decreases. This explains the increase in the nondimensional natural frequency as the nanotube radius decreases lower than 3 nm, as shown in Fig. 5. Per the previous discussion, the first mechanism is dominant as the nanotube radius decreases from 10 to 3 nm while the second mechanism comes into play as the nanotube radius decreases lower than 3 nm.

It can also be observed from Fig. 5 that the effect of the applied pressure on the nondimensional natural frequencies of the nanotube is enhanced as the nanotube size decreases. As shown in the figure, the nanotube exhibits nearly the same nondimensional natural frequencies, even if the applied pressure is increased from 2 to 20 MPa, when the nanotube radius is bigger than 10 nm. However, the nondimensional natural frequencies significantly depend on the applied pressure when the nanotube radius is smaller than 10 nm.

## Conclusion

An accurate prediction of dynamics of water conveying nanotubes is contingent upon an accurate prediction of the viscosity of water that encompasses wettability and confinement size effects. We utilized a hybrid continuum-molecular mechanics (HCMM) to determine water viscosity variations in nanotubes. Nanotubes of different surface wettability along with various radii were simulated for range of operational pressures to analyze the first four non-dimensional natural frequencies of water conveying nanotubes. Two different bifurcations were observed where mode divergence instability and flutter instability occurred. It was revealed that the activation of these two instabilities depends on the nanotube size and wettability. The critical pressure value at which a nanotube exhibits instability would increase/decrease as the nanotube size decreases, and it increases as the nanotube hydrophilicity increases. In addition, it was demonstrated that, under the same applied pressure, hydrophilic nanotubes are more stable than hydrophobic nanotubes. Therefore, hydrophilic nanotubes are preferred over hydrophobic nanotubes in applications where operational pressures would deviate from a nominal pressure value. The findings of the present study show that the pressure dependence of the nanotube stability is unpredictable unless careful calculations are made based on the developed model.

## Supplementary information

Supplementary Information.

## Data Availability

The data that support the findings of this study are included in the article. Any further requested information can be addressed to the corresponding author.
